# Hypothyroidism Complicating Nephropathy in a Diabetes Patient

**DOI:** 10.1155/2012/237563

**Published:** 2012-12-10

**Authors:** Jayaprakash Sahoo, Ilangovan Veerappan, Anila Abraham, Somasundaram Hariharan

**Affiliations:** ^1^Department of Endocrinology, Pondicherry Institute of Medical Sciences, Puducherry 605014, India; ^2^Department of Nephrology, Pondicherry Institute of Medical Sciences, Puducherry 605014, India; ^3^Department of Pathology, Madras Medical Mission, Chennai 600037, India

## Abstract

We describe a patient with type 2 diabetes mellitus and autoimmune hypothyroidism who presented with elevated serum creatinine possibly due to subclinical rhabdomyolysis induced by hypolipidemic drug therapy in the background of diabetic nephropathy. Both hypothyroidism and rhabdomyolysis were asymptomatic in this case as evidenced by lack of classical clinical features of hypothyroidism despite elevated serum TSH and absent pigment cast in renal biopsy. The combination of diabetes mellitus and hypothyroidism is common in the general population and should not be forgotten in patients with diabetes and kidney disease.

## 1. Introduction 

 Type 2 diabetes mellitus is associated with various components of metabolic syndrome like obesity, hypertension, and dyslipidemia [1]. Hypothyroidism also leads to secondary dyslipidemia [2]. Drugs used for treatment of dyslipidemia like statins and fibrates cause myalgia, myopathy, and rhabdomyolysis with renal injury particularly in the presence of hypothyroidism [3]. This case report discusses the above complications in a diabetes patient with primary hypothyroidism and also the renal response to thyroxine supplementation. 

## 2. Case Report

A forty-year-old male having diabetes mellitus for six years came with complaints of pain in both lower limbs. He was neither a smoker nor an alcoholic and had family history of diabetes mellitus with chronic kidney disease. He was a teacher by occupation with sedentary lifestyle and was compliant with diabetic diet. There was no history of hypertension, coronary artery disease, transient ischemic attacks/cerebrovascular accident, and recurrent urinary tract infection, and he was not evaluated for microvascular complications like nephropathy/retinopathy. He was denying the intake of any nephrotoxic drug in the past. Glycemic control was fair (HbA_1c_, 6.7%) with oral hypoglycaemic agents (one gram of Metformin and one milligram of Glimepiride). On examination, he was overweight (BMI, 24) with sensory neuropathy (lower limbs > upper limb) and blood pressure of 110/70 mmHg with palpable peripherals pulses. There was neither goiter nor any sign of hypothyroidism.

 The patient had normal reports of ECG, 2D echocardiography, and arterial doppler of lower limbs. But the lipid profile and renal function test were deranged (Table 1). We added Pregabalin (75 mg) for sensory neuropathy and Atorvastatin (10 mg) and Fenofibrate (145 mg) for dyslipidemia. The patient came after four and half months with reports that showed primary hypothyroidism due to Hashimoto's thyroiditis (Table 1) with urine spot protein creatinine ratio of 1.6 and normal ultrasound findings of kidneys/ureter/bladder. There was no evidence of diabetic retinopathy. But additionally, there was elevated serum aspartate aminotransferase (SGOT) level. Hypolipidemic drug complicating hypothyroidism-induced muscle damage was suspected and was confirmed by increased serum creatine phosphokinase (CPK) and lactate dehydrogenase (LDH) level. But there was no history of myalgia, myopathy, decrease in urine output, or cola-coloured urine. The hypolipidemic drugs were stopped, and he was started on thyroxine supplementation.

**Figure 1:**
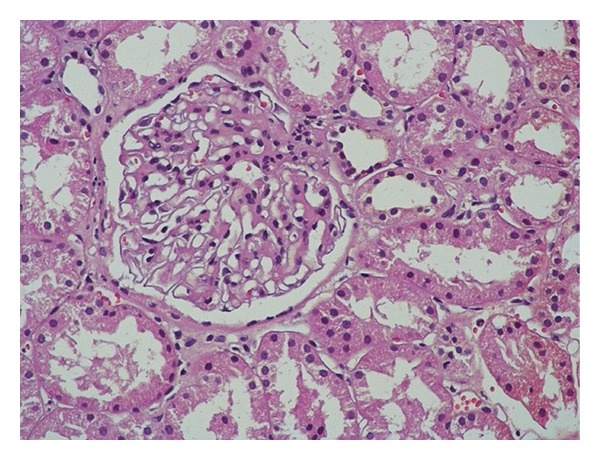
Renal biopsy showing mild mesangial expansion.

 A renal biopsy was done due to abnormal renal function, proteinuria in the absence of retinopathy, and a positive family history of chronic kidney disease. The glomeruli were normocellular with mildly thickened basement membranes and mild mesangial matrix expansion (Figure 1). There were no spikes or duplication of the basement membrane. The tubular epithelial cells showed cytoplasmic vacuolization and sloughing. There was no pigment casts seen. There was focal interstitial fibrosis and tubular atrophy involving about 20% of the core. There was arteriolar hyalinosis and mild medial hyperplasia of the arteries. The features were consistent with early diabetic nephropathy (class-IIa) [4]. The electron microscopy showed mild-to-moderate basement membrane thickening with mild segmental mesangial expansion without any evidence of immune complex deposition. Additionally, there was prominent diffuse isometric tubular cytoplasmic vacuolization.

The patient was started on Telmisartan (20 mg) with periodic monitoring of serum creatinine, potassium, and urine routine/microscopic examination. Repeat investigations (Table 1) revealed normal CPK, LDH, and SGOT with improving serum creatinine and proteinuria. 

## 3. Discussion

 Severe hypothyroidism (biochemically) may be completely asymptomatic. Though our patient had TSH level more than 100 mIU/L, he did not have any symptoms of hypothyroidism. Jarløv et al. had reported earlier that diagnosis of hypothyroidism depends on laboratory parameters rather than clinical findings [5]. In an analysis of patients with varying degrees of hypothyroidism, Zulewski et al. highlighted the discordance between biochemical and clinical hypothyroidism. Some patients with severe biochemical hypothyroidism had only mild clinical manifestations, whereas other patients with trivial biochemical changes had quite severe physical signs and symptoms [6]. So, it appears that there can be complete dissociation between the biochemical hypothyroidism and tissue hypothyroidism at the peripheral target organs in an individual patient.

 Hypolipidemic drugs do not precipitate rhabdomyolysis in all hypothyroid patients. The exact incidence and the mechanisms are not clear [3]. The total CPK was elevated and the SGOT : SGPT ratio was more than three, both suggesting muscle injury in this case. The muscle injury was not of significant degree to precipitate pigment-induced acute tubular injury as evidenced by relatively stable serum creatinine in the period when the patient was taking the hypolipidemic drugs. Impaired glycogenolysis and mitochondrial oxidative metabolism are possible mechanisms for rhabdomyolysis in hypothyroid patients [7]. The theory behind statin-induced muscle injury is skeletal muscle cell membrane instability due to reduced small GTP (guanosine 5′- triphosphate) binding proteins and cholesterol synthesis, which is exaggerated by addition of fibrates [8]. Classically, rhabdomyolysis is associated with muscle pain and weakness with increased muscle enzymes in the serum [9]. But it may be totally asymptomatic like the index case. The only clue for muscle damage in our patient was increased isolated serum SGOT level, which was confirmed by increased level of other muscle enzymes (CPK & LDH). 

 There are multiple reasons for reduction in GFR in patients with hypothyroidism: reduced renal blood flow [10], increased peripheral vascular resistance [11], and intrarenal vasoconstriction due to reduced renal response to vasodilators [12, 13]. There is also reduced expression of renal vasodilators like vascular endothelial growth factor (VEGF) and insulin-like growth factor-1 (IGF-1) [14]. Additionally, there is decreased renin release [15] and impaired renin angiotensin aldosterone system activity which contributes to reduced GFR [16]. It has been reported that there is decrease in tubular secretion of creatinine—a process mediated by the thyroid hormones via the Na^+^/Ca^2+^ exchanger and the Na^+^/K^+^ ATPase activity further contributing to increase in serum creatinine [17]. Additionally, hypothyroidism leads to changes similar to early diabetic nephropathy like thickening of glomerular and tubular basement membrane with expanded mesangial matrix which is responsible for glomerular leaking of proteins [18–20].

 Within one month after starting thyroxine supplementation along with withdrawal of the hypolipidemic drugs, the serum creatinine decreased from 1.6 mg/dL to 1.1 mg/dL with some improvement in proteinuria (Table 1). However, serum creatinine and proteinuria remained static for next 3 months in spite of continuation of thyroxine replacement. The initial improvement in renal parameters was due to both correction of rhabdomyolysis and hypothyroidism. The residual renal dysfunction may be due to presence of underlying diabetic nephropathy. But the absence of retinopathy in this case makes diabetic nephropathy less likely as a cause for renal dysfunction, which will be confirmed during further followup. 

## 4. Conclusion 

 The correlation between serum TSH, tissue hypothyroidism, and clinical features is poor. Hypothyroidism-induced rhabdomyolysis with or without associated statin/fibrate therapy may lead to abnormal renal function in a diabetes patient. Additionally, hypothyroidism also causes renal changes similar to early diabetic nephropathy. This should be kept as one differential diagnosis for reversible renal dysfunction in a diabetes patient.

## Figures and Tables

**Table 1:** Investigations at different time points.

	−4.5 months	0 time	+1 month	+4 months	Reference
Serum TSH	—	160	09	04	0.27–4.2 mIU/L
TPO antibody	—	>500	—	—	<40 IU/L
Total cholesterol	381	220	254	259	<200 mg%
Triglyceride	1286	250	619	280	<150 mg%
LDL	—	119	—	148	<100 mg%
HDL	16	51	26	55	>40 mg%
HbA1c	6.7%	7.2%	—	—	6%-7%
Serum creatinine	1.5	1.6	1.1	1.1	0.6–1.2 mg%
*MDRD eGFR	55	51	79	79	≥90 mL/min per 1.73 m^2^
Serum CPK	—	2810	104	—	25–200 IU/L
Serum LDH	—	549	378	—	225–420 IU/L
Serum SGOT	—	201	15	—	<37 IU/L
Serum SGPT	—	66	—	—	<65 IU/L
Serum K^+^	—	5.5	4.4	4.3	3.5–5 meq/L
Serum albumin	—	4.2	4.2	4.0	4-5 gm%
Urine protein/creatinine ratio	—	1.6	1.0	1.0	<0.3
Urine microscopy	—	4-5 pus cells/HPF	8–10 pus cells/HPF	4–6 pus cells/HPF	0–2 pus cell/HPF
Urine C/S	—	Sterile	Sterile	—	Sterile

∗MDRD eGFR: Modification of diet in renal disease estimated glomerular filtration rate.
